# Pseudohyperkalemia - Potassium released from cells due to clotting and centrifugation - a case report

**DOI:** 10.11613/BM.2018.011002

**Published:** 2018-01-10

**Authors:** Tomáš Šálek

**Affiliations:** 1 Department of biomedical sciences, Medical Faculty of the University of Ostrava, Ostrava – Zábřeh, Czech Republic; 2 Department of Clinical biochemistry and pharmacology, Tomas Bata hospital in Zlín, Zlín, Czech Republic

**Keywords:** hyperkalemia, case report, electrolytes, pseudohyperkalemia

## Abstract

Hyperkalemia is a potentially lethal condition. Pseudohyperkalemia should be always excluded before implementing treatment to prevent inappropriate cause of hypokalemia – equally a potentially lethal condition. Here we present a case report of a 62 year female with chronic myeloproliferative disorder, *i.e.* essential thrombocythemia. The laboratory test results for potassium concentration were 6.3 mmol/L, for platelet count 1305 x10^9^/L and for leukocyte count 39.8 x10^9^/L. This was due to a temporary drug withdrawal after a surgical intervention for gastric bleeding. Potassium concentration in lithium heparin plasma collected in a vacuum tube without gel separator and in whole blood syringe were 4.6 mmol/L and 3.4 mmol/L, respectively. It means that mechanical stress such as centrifugation can contribute to spurious hyperkalemia. Prior to reporting unexpected hyperkalemia result, pseudohyperkalemia should always be considered by the laboratory. Such potassium results require investigation in case it is pseudohyperkalemia, which may be due to thrombocytosis and leukocytosis. In cases where thrombocytosis or leukocytosis exists, an interpretative comment indicating these conditions inserted with the results of the potassium concentration can increase awareness for more accurate patient care decisions.

## Introduction

When patients’ results are interpreted, the total testing process which includes pre-analytical, analytical and post-analytical phases should be taken into account (*1*).

Hyperkalemia is a potentially lethal condition. Differential diagnosis is broad and includes decreased glomerular filtration rate, acidemia, drugs interfering with potassium metabolism (*e.g.* digoxin) and spurious hyperkalemia due to pre-analytical conditions (*2*).

In some cases a problem in the pre-analytical phase can be recognized during the interpretation of laboratory test results (*3*). Pseudohyperkalemia due to thrombocytosis or leukocytosis is an example of such situation. Pseudohyperkalemia should be considered whenever increased platelets or leukocytes in blood count coincide with unexplained serum hyperkalemia, typically in a patient who is without renal failure or acidosis or who does not take drugs such as angiotensin converting enzyme inhibitors and cardiac glycosides (*4*).

Pseudohyperkalemia can be confirmed by determining plasma potassium in vacuum tubes with lithium heparin after centrifugation, and/or by whole blood potassium determination in electrolyte balanced lithium heparin syringe (*5*).

Pseudohyperkalemia should only be considered when the serum potassium concentration exceeds that of plasma by at least 0.4 mmol/L (*6*). To reduce inappropriate treatment of hyperkalemia the difference of 1.0 mmol/L between serum and plasma was found practical. Whole blood is recommended for potassium measurement (*7*).

Mechanical stress such as centrifugation can lead to spurious hyperkalemia in patients with chronic lymphocytic leukaemia or acute myeloid leukaemia due to increased fragility of their white blood cells (*8*). The added value of this case report is showing the difference in results between these two possible ways of confirming pseudohyperkalemia.

## Case report

### Informed consent

The patient gave informed consent to all relevant and related procedures in a form of a handwritten signature before the procedures were performed. The publication of this case report was approved by the Tomas Bata hospital Ethics committee.

### Case and methods description

A 62 years old woman was urgently admitted to the surgery department for bleeding from gastric ulcer with haemorrhagic shock. She was successfully operated.

Her chronic medical history included diabetes mellitus, chronic myeloproliferative disorder of essential thrombocythemia type with the presence of Janus kinase 2 V617F mutation, and chronic hepatic disease, probably non-alcoholic fatty liver disease. She was regularly taking hydroxylcarbamide 500 mg daily and insulin. The hydroxycarbamide medication was discontinued because of gastrointestinal bleeding.

Her blood count, electrolytes, kidney function and other biochemistry laboratory tests were taken 12 days after the surgery. The tests revealed serum hyperkalemia of 6.3 mmol/L. Blood count revealed leukocytosis and thrombocytosis (Table 1). Her estimated glomerular filtration rate was decreased. She received transfusions during the operation. Laboratory tests were measured on a daily basis.

**Table 1:** The patient’s laboratory results from different sample types

**Test**	**Results**			**Reference range**
**Serum**	**Whole blood**	**Li-heparin plasma**
Serum free haemoglobin (g/L)	0.09		0.00	0.00 – 0.25
Glucose (mmol/L)	5.5	10.2	/	3.9 – 5.5 (whole blood, plasma); 3.8 – 5.5 (serum)
Sodium (mmol/L)	131	128	132	136 – 144
Potassium (mmol/L)	6.3	3.4	4.6	3.8 – 5.1 (serum); 3.5 – 4.8 (whole blood, plasma)
Chloride (mmol/L)	99	104	104	95 – 107
Urea (mmol/L)	7.5	/	/	2.0 – 6.7
Creatinine (µmol/L)	114	/	/	49 - 90
eGFR (mL/s/1.73m^2^)	0.74	/	/	1.50 – 2.50
Albumin (g/L)	34.4	/	/	35.0 – 52.0
C-reactive protein (mg/L)	22	/	/	< 3
White blood cells (x10^9^/L)	39.8	/	/	4.0 – 10.0
Red blood cells (x10^12^/L)	4.34	/	/	3.80 – 5.20
Platelets (x10^9^/L)	1305	/	/	150 – 400
Whole blood pH	/	7.421	/	7.360 – 7.440
Whole blood pCO_2_ (kPa)	/	3.72	/	4.60 – 6.00
Whole blood pO_2_ (kPa)	/	11.60	/	10.67 - 14.40
Base excess (mmol/L)	/	- 5.8	/	- 2.5 – 2.5
Measured saturation of haemoglobin (%)	/	96.7	/	95.0 – 99.0
eGFR - estimated glomerular filtration rate using the Chronic Kidney Disease – Epidemiology Collaboration (CKD-EPI) equation.				

Gradual increase in both serum potassium concentration and platelet number was observed after surgery. Platelets increased from 245 to 1392 x10^9^/L. Serum potassium concentration increased from 4.7 to 6.4 mmol/L. The correlation between serum potassium concentration and platelet count was found.

Our department has two checkpoint levels in reporting laboratory test results. At first level, a biomedical scientist evaluates the effect of haemolysis, lipaemia and icterus, which are measured automatically in all samples. Serum potassium concentration was not affected by these variables. Electrolytes were measured repeatedly within few minutes with the same results in the same sample. At the second checkpoint, the clinical plausibility of all laboratory results is assessed. An interpretative comment was added in this case: “Pseudohyperkalemia due to thrombocytosis and leukocytosis is suspected. New sampling to lithium heparin tube or a measurement of plasma electrolytes in syringe for a blood gas analysis is recommended.”. This information was also phoned to the surgery department and the information about the phone call was recorded in the laboratory information system.

Both determinations were performed and confirmed spurious hyperkalemia. Results are shown in Table 1.

### Laboratory analyses

VACUETTE^®^ red top 6 mL tube (Greiner Bio-One GmbH, catalogue number 476092, Kremsmunster, Austria) with clot activator and without gel separator was used for venous blood collection. The separation of cells from serum was performed within 1 hour after collection. The sample was centrifuged for 10 minutes at 1500xg. Serum potassium concentration and other serum tests were measured on ci16200 Abbott Architect analyser (Abbott Laboratories, Illinois, USA).

Whole blood pH, pCO_2_, pO_2_, glucose and electrolytes tests were performed on Radiometer ABL 800 FLEX blood gas analyser (Radiometer, Bronshoj, Denmark) by electrochemistry methods. The potentiometric principle is applied in the measurement of pH, pCO_2_ and electrolytes. The amperometry (Clark electrode) is applied in measurement of pO_2_ and glucose. This is the point of care testing analyser which is not present in the central laboratory.

An anaesthesiologist performed the blood collection from left femoral artery to Radiometer dry electrolyte-balanced lithium heparin syringe (catalogue number 956-552 PICO 50). The tests were measured within 5 minutes after collection. This sample was not checked for haemolysis. VACUETTE^®^ green top Lithium Heparin coated 3 mL tube without gel separator (Greiner Bio-One GmbH, catalogue number 454082, Kremsmunster, Austria) was used for venous blood collection. The separation of cells from plasma was performed within 15 minutes after collection (the sample was taken to the laboratory immediately after collection). The sample was centrifuged for 10 minutes at 1500xg. Plasma electrolytes were measured on ci16200 Abbott Architect analyser (Abbott Laboratories, Illinois, USA). Blood count was measured on COULTER® LH 750 haematology analyser (Beckman Coulter, Brea, USA).

All 3 samples were taken in a lying position. The first and the third ones were delivered to the laboratory manually. Only the first sample was taken in fasting state. It explains the elevated plasma glucose in a diabetic patient after meal. The first sample was accepted by laboratory at 8:10 a.m. and reported at 9:19 a.m. The communication with clinicians started at 9:19 a.m. The next sample was collected at 9:34 a.m. and reported at 9:37 a.m. (point of care testing analyser). This second sample confirmed pseudohyperkalemia.

### Results

The patient had a combination of laboratory test results of serum hyperkalemia, thrombocytosis and leukocytosis. Potassium in plasma was lower when measured in vacuum tube coated with lithium heparin and lowest in whole blood. All results are included in Table 1.

A correlation between serum potassium concentration and platelet count was found. The Spearman rank correlation coefficient of 11 consecutive paired values after surgery was 0.64 (P < 0.05). Data are presented in Table 2.

**Table 2:** Serum potassium concentration and platelet count after surgery

**Sampling date**	**Serum potassium (mmol/L)**	**Thrombocytes (x10^9^/L)**	**Hydroxycarbamide treatment**
24.7.2017.	4.7	245	No
25.7.2017.	4.6	321	No
26.7.2017.	4.5	404	No
27.7.2017.	4.9	479	No
28.7.2017.	5.8	294	No
29.7.2017.	6.2	509	No
30.7.2017.	4.9	549	No
1.8.2017.	6.3	1305	Yes
2.8.2017.	6.4	1392	Yes
3.8.2017.	5.2	831	Yes
9.8.2017.	4.0	450	Yes

### What happened?

This report demonstrated a case of pseudohyperkalemia due to a combination of thrombocytosis and leukocytosis in a patient with essential thrombocythemia. The concentration of potassium was much higher in serum than in plasma and whole blood. Centrifuged plasma showed higher potassium concentration than whole blood. It may be assumed with high probability that centrifugation can also lead to cell destruction and a consequent increase in plasma potassium.

## Discussion

The novelty of this case report consists in finding lower concentration of potassium in whole blood than in lithium heparin plasma after centrifugation. It may be explained by the fact that centrifugation can cause breakdown of some cells, which in turn results in increased plasma potassium concentration.

Laboratory scientists generally have three options when dealing with elevated potassium. The first option is to review the serum indices, haemolysis specifically. In this case the haemolysis was not present. The second option is to look for pathophysiological reasons and the third option is to consider pseudo reasons. The final decision on what potassium result is reported may require a consultation with clinical staff along with an interpretative comment to aid clinical staff.

Pre-analytical phase is a continuous challenge for laboratory professionals. Haemolysis is the most frequent pre-analytical issue (*9*).

Potassium is also released from thrombocytes during blood clotting. It explains higher potassium concentration in serum than in plasma even in healthy subjects. This difference is higher than 0.4 mmol/L in patients with thrombocytosis (*6*). This is consistent with our results. If the whole blood sample is used, the proposed cut-off to avoid inappropriate treatment is 1 mmol/L (*7*). Leukocytosis is one of the causes of pseudohyperkalemia (*10*). Leukocytosis probably contributed to pseudohyperkalemia also in our patient.

Hyperkalemia is a common complication in transfusion of stored blood in critically ill patients (*11*). Haemolysis-caused pseudohyperkalemia in whole blood samples can also be measured by acid base analysers (*12*).

Our department has an internal policy document named “Reporting laboratory test results”. It specifies two checks of laboratory test results. An interpretative comment is advised for the second check, if suitable. In the case of hyperkalemia, the comment should primarily include evaluation of renal function tests and blood count (number of leukocytes and thrombocytes) (*13*, *14*). That is why it is practical to add the interpretative comment in special situations. Such a comment explains the situation and recommends further diagnostic algorithm.

The major limitation of this work is that only a single patient was included.

## What YOU should / can do in your laboratory to prevent such errors.

Thrombocytosis and extreme leukocytosis lead to pseudohyperkalemia in serum.
Measurement of potassium in plasma confirms this condition.
Reporting test results extended with an interpretative comment including a recommendation of further diagnostic algorithm is helpful and has an important educational role.
Centrifugation may contribute to pseudohyperkalemia in some cases.
It is helpful to measure potassium in whole blood sample in evaluation of pseudohyperkalemia.
